# Data on optimization and drug release kinetics of nanostructured lipid carriers containing ondansetron hydrochloride prepared by cold high-pressure homogenization method

**DOI:** 10.1016/j.dib.2019.104475

**Published:** 2019-09-05

**Authors:** Van-An Duong, Thi-Thao-Linh Nguyen, Han-Joo Maeng, Sang-Cheol Chi

**Affiliations:** College of Pharmacy, Gachon University, Yeonsu-gu, Incheon, 21936, South Korea

**Keywords:** Nanostructured lipid carriers, Ondansetron hydrochloride, Cold high-pressure homogenization, Particle size, Polydispersity index

## Abstract

Nanostructured lipid carriers (NLCs), the second generation of lipid nanoparticles could enhance the drug loading capacity and minimize the drug expulsion during storage [1,2]. They are prepared from mixtures of solid and liquid lipids [3,4]. The article described the data for the preparation, optimization, and drug release studies of NLCs loaded with ondansetron hydrochloride (OSH), a water-soluble drug. The OSH-loaded NLCs were prepared using a modified cold high-pressure homogenization method. The NLCs were optimized for various parameters of formulation and preparation process on the basis of particle size (PS), polydispersity index (PI), entrapment efficiency (EE), and drug loading (DL). The dataset presented here supports “Nanostructured lipid carriers containing ondansetron hydrochloride by cold high-pressure homogenization method: Preparation, characterization, and pharmacokinetic evaluation” [5].

**Table:** Specifications Table

Subject area	*Chemistry, Biology, Pharmaceutical Sciences*
More specific subject area	*Lipid nanoparticles*
Type of data	*Table and Figure*
How data was acquired	*Particle size analyzer (Zetasizer Nano-S90, Malvern, UK) for PS and PI. HPLC (1260 Infinity, Agilent Technologies, CA, USA) for quantification (solubility, EE, and DL). FTIR spectrophotometer (Tensor 27, Bruker. MA, USA).*
Data format	*Raw*
Experimental factors	*Formulation and process parameters were varied for optimization of PS, PI, EE, and DL.*
Experimental features	*Various formulations were prepared by a modified cold high-pressure homogenization method to obtain NLCs. Drug release data were fitted to various kinetic models.*
Data source location	*College of Pharmacy, Gachon University, Yeonsu-gu, Incheon, South Korea*
Data accessibility	*Data are presented in this article.*
Related research article	*Van-An Duong, Thi-Thao-Linh Nguyen, Han-Joo Maeng, and Sang-Cheol Chi, Nanostructured lipid carriers containing ondansetron hydrochloride by cold high-pressure homogenization method: Preparation, characterization, and pharmacokinetic evaluation, Journal of Drug Delivery Science and Technology, Volume 53, 2019, 101,185, DOI:**https://doi.org/10.1016/j.jddst.2019.101185*

**Table:** 

**Value of the data** • The data summarize the effect of different parameters of formulation on PS, PI, EE, and DL of NLCs, which can be useful for other researchers working on lipid nanoparticles. • The influences of high-pressure homogenization on NLCs are presented. • Data of drug release kinetics can be used to investigate the release mechanism of the drug from NLCs.

## 1 Data

**Fig. 1:**
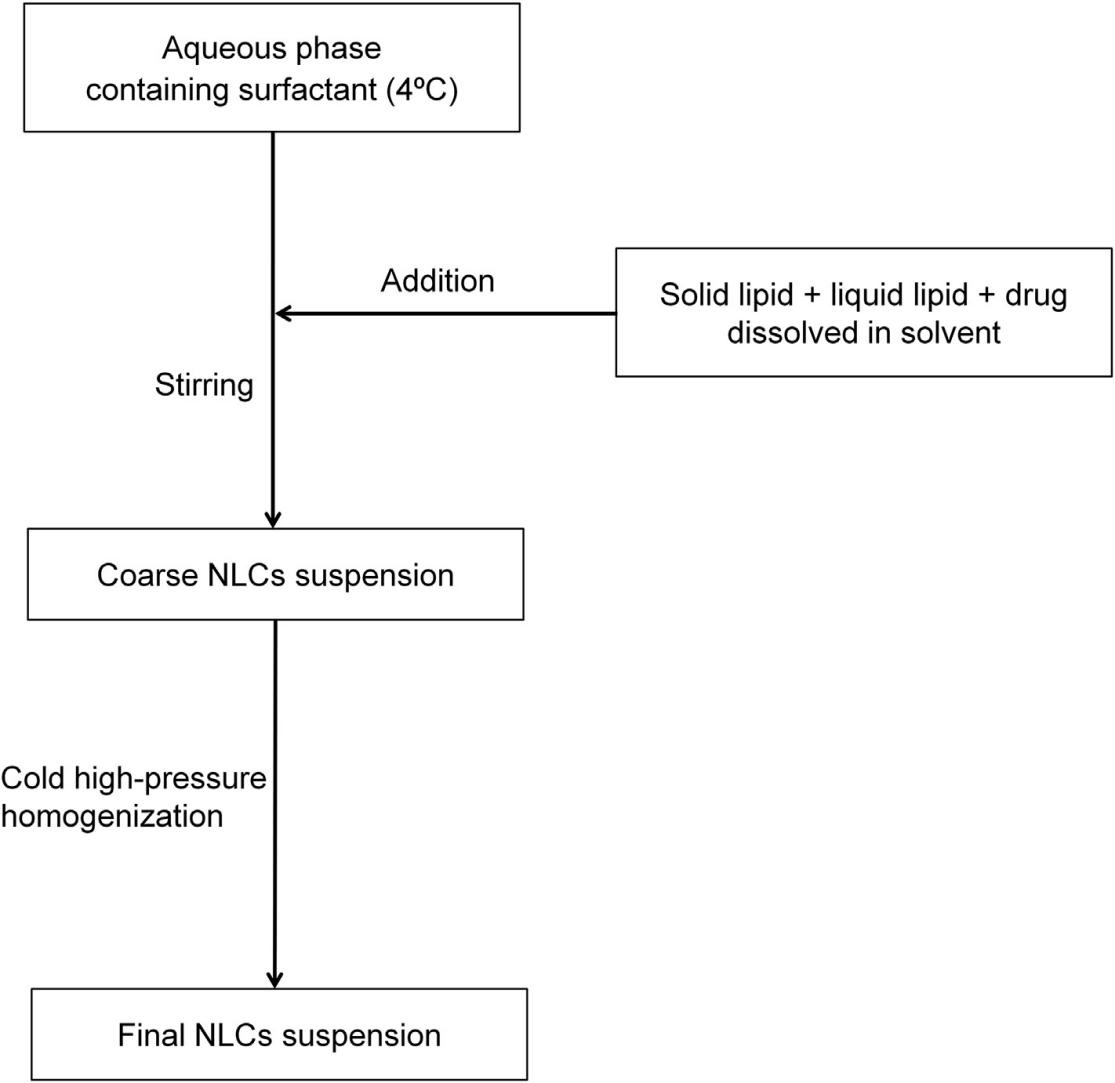
Flow chart representing the preparation of nanostructured lipid carriers by a modified cold high-pressure homogenization method.

**Fig. 2:**
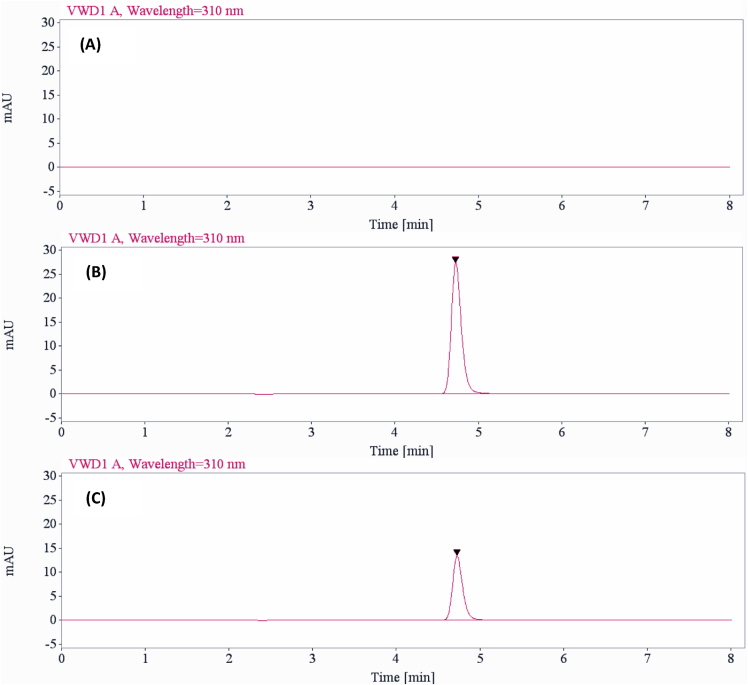
Chromatograms of blank sample (A), OSH standard solution (B), and sample from release study (C).

**Fig. 3:**
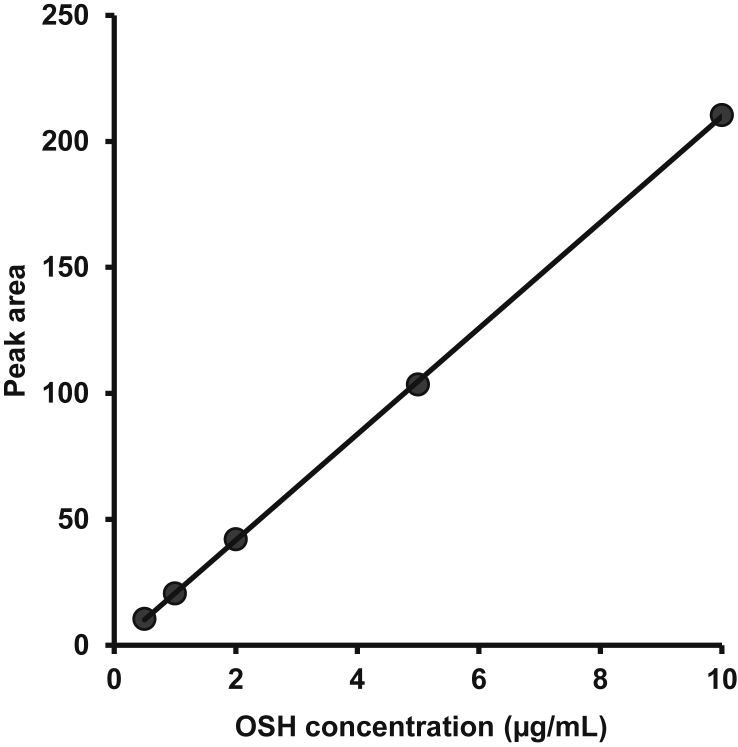
Calibration curve for OSH analysis using HPLC method.

**Fig. 4:**
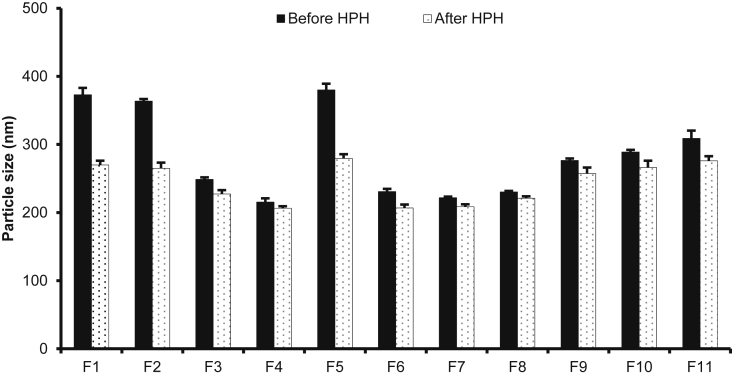
Effect of high-pressure homogenization (HPH, 500 bars × 6 cycles) on particle size of NLCs (Mean ± SD, n = 3).

**Fig. 5:**
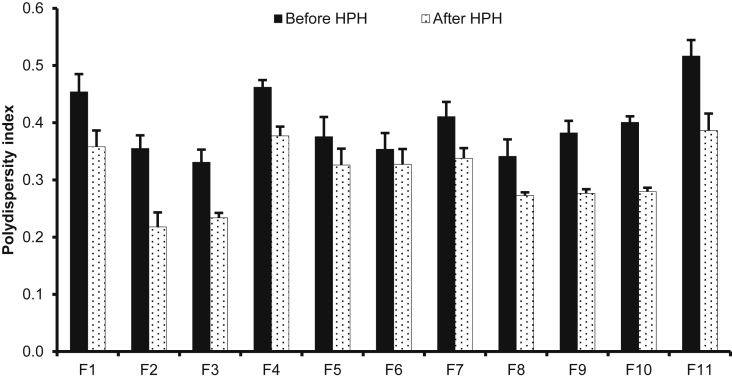
Effect of high-pressure homogenization (HPH, 500 bars × 6 cycles) on polydispersity index of NLCs (Mean ± SD, n = 3).

**Fig. 6:**
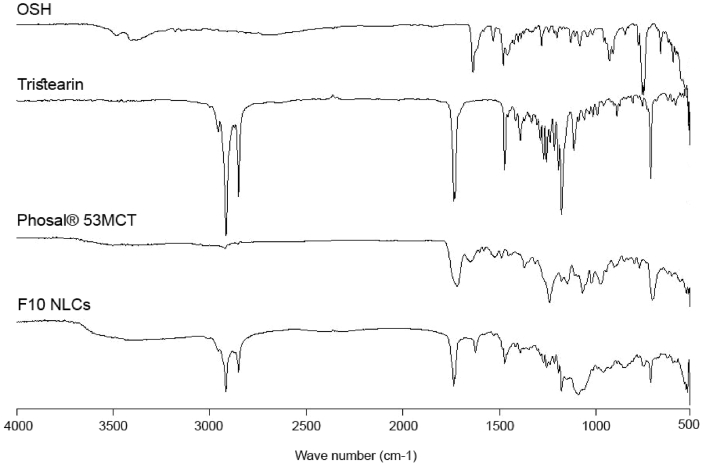
FTIR spectra of OSH, tristearin, Phosal® 53MCT, and lyophilized F10.

Fig. 1 shows the preparation process of NLCs using a modified high-pressure homogenization method. Fig. 2, Fig. 3, Table 1A, Table 1BA and 1B presents validation data of the HPLC method for OSH analysis. Table 2 represents the solubility of OSH in various liquid oils, which can be used to select the proper one for NLCs preparation, since a liquid lipid with a higher drug solubility may result in a better drug loading capacity [1], [2], [3], [4], [5], [6], [7]. Various parameters of the formulation were optimized on the basis of their influences on PS, PI, EE, and DL. These parameters included pH of the aqueous phase (Table 3), solvent (Table 4A) and solid lipid (Table 4B). Table 5, Fig. 4, Fig. 5 show PS and PI of different formulations before and after homogenization, which can be useful to evaluate the effects of homogenization on NLCs. Fig. 6 presents FTIR spectra of OSH, tristearin, Phosal® 53MCT, and F10 NLCs formulation. *In vitro* release data were fitted to various kinetic models. Table 6, Table 7 exhibit parameters of these kinetic models after fitting. The results can be valuable to investigate the release mechanism of drug from NLCs.

**Table 1A:** Relationship between OSH concentration and HPLC peak area for establishment of calibration curve.

Nominal concentration (μg/mL)	Peak area
0.5	10.43
1	20.61
2	42.12
5	103.52
10	210.38

**Table 1B:** Intra-day and inter-day precision and accuracy of OSH assay.

Nominal concentration (μg/mL)	Intra-day		Inter-day	
	Precision (%)	Accuracy (%)	Precision (%)	Accuracy (%)
0.5	0.86	101.88	2.84	102.62
1	0.57	99.44	1.61	99.63
2	1.07	99.86	0.43	99.35
5	0.88	98.06	0.96	99.26
10	0.76	99.65	0.74	99.65

**Table 2:** Drug solubility in liquid lipids.

Liquid lipids	Solubility (mg/g)
Capryol® 90	2.39 ± 0.06
Labrasol®	15.7 ± 2.4
Lauroglycol® 90	2.47 ± 0.41
Oleic acid	1.25 ± 0.05
Peceol®	6.29 ± 1.70
Phosal® 53MCT	64.7 ± 5.5
Sesame oil	6.41 ± 1.02

**Table 3:** Effects of pH on PS, PI, EE, and DL of NLCs.

Formulation	pH	PS (nm)	PI	EE (%)	DL (%)
A1	Water	176 ± 2	0.19 ± 0.01	40.5 ± 7.7	3.89 ± 0.71
A2	2	187 ± 1	0.22 ± 0.01	31.9 ± 3.3	3.09 ± 0.31
A3	4	297 ± 13	0.30 ± 0.00	40.1 ± 2.3	3.86 ± 0.21
A4	6	230 ± 1	0.29 ± 0.01	47.8 ± 5.4	4.56 ± 0.49
A5	7.4	249 ± 3	0.33 ± 0.02	93.1 ± 0.3	8.52 ± 0.02
A6	8	406 ± 4	0.58 ± 0.02	94.3 ± 1.0	8.62 ± 0.08
A7	10	323 ± 6	0.59 ± 0.05	94.3 ± 0.8	8.62 ± 0.07
A8	12	390 ± 13	0.63 ± 0.06	94.1 ± 1.6	8.60 ± 0.13

Composition of all the formulations was of tristearin: Phosal® 53MCT: OSH (60:40:10, w/w).

**Table 4A:** Effects of solvent on PS and PI of NLCs.

Formulation	Lipid composition^a^ (w/w)	Solvent	PS (nm)	PI
B1	80:20	Ethanol	367 ± 7	0.29 ± 0.01
B2		Acetone	347 ± 5	0.39 ± 0.05
B3		Isopropanol	524 ± 5	0.34 ± 0.01
B4	60:40	Ethanol	307 ± 8	0.29 ± 0.02
B5		Acetone	310 ± 9	0.28 ± 0.03
B6		Isopropanol	474 ± 8	0.25 ± 0.01
B7	40:60	Ethanol	273 ± 9	0.29 ± 0.02
B8		Acetone	294 ± 3	0.25 ± 0.01
B9		Isopropanol	388 ± 10	0.27 ± 0.01

a Precirol® ATO 5: Phosal® 53 MCT.

**Table 4B:** Effects of solid lipid on PS and PI of NLCs.

Formulation	% Phosal® 53 MCT	Solid lipid	PS (nm)	PI
C1	20	Tristearin	364 ± 3	0.36 ± 0.02
C2	20	Precirol® ATO 5	367 ± 7	0.29 ± 0.02
C3	40	Tristearin	249 ± 3	0.33 ± 0.02
C4	40	Precirol® ATO 5	307 ± 8	0.29 ± 0.02
C5	60	Tristearin	215 ± 6	0.46 ± 0.01
C6	60	Precirol® ATO 5	273 ± 9	0.29 ± 0.02

**Table 5:** Effect of high-pressure homogenization (HPH, 500 bars × 6 cycles) on particle size and polydispersity index of NLCs (Mean ± SD, n = 3).

Formulation	Composition^a^	Polysorbate 80 (%)	Before HPH		After HPH	
			PS (nm)	PI	PS (nm)	PI
F1	100: 0: 10	0.5	373 ± 10	0.45 ± 0.03	270 ± 6	0.36 ± 0.03
F2	80: 20: 10	0.5	364 ± 3	0.36 ± 0.02	265 ± 8	0.22 ± 0.03
F3	60: 40: 10	0.5	249 ± 3	0.33 ± 0.02	227 ± 6	0.23 ± 0.01
F4	40: 60: 10	0.5	215 ± 6	0.46 ± 0.01	206 ± 3	0.38 ± 0.02
F5	60: 40: 10	0.1	380 ± 9	0.38 ± 0.03	280 ± 6	0.33 ± 0.03
F6	60: 40: 10	1	231 ± 4	0.35 ± 0.03	207 ± 5	0.33 ± 0.03
F7	60: 40: 10	1.5	222 ± 2	0.41 ± 0.03	208 ± 4	0.34 ± 0.02
F8	60: 40: 7.5	0.5	230 ± 1	0.34 ± 0.03	221 ± 3	0.27 ± 0.01
F9	60: 40: 12.5	0.5	277 ± 3	0.38 ± 0.02	257 ± 6	0.28 ± 0.01
F10	60: 40: 15	0.5	289 ± 3	0.40 ± 0.01	266 ± 10	0.28 ± 0.01
F11	60: 40: 17.5	0.5	309 ± 12	0.52 ± 0.03	276 ± 7	0.39 ± 0.03

a Tristearin: Phosal® 53MCT: OSH (w/w).

**Table 6:** Parameters of various kinetic models after fitting release data of different NLCs.

	Zero-order		First-order		Higuchi		Hixson-Crowell	
	R^2^	k	R^2^	k	R^2^	k	R^2^	k
F1	0.725 ± 0.048	2.78 ± 0.03	0.988 ± 0.001	0.087 ± 0.007	0.948 ± 0.006	16.4 ± 0.3	0.998 ± 0.001	0.025 ± 0.002
F2	0.856 ± 0.024	2.42 ± 0.03	0.995 ± 0.001	0.057 ± 0.003	0.957 ± 0.004	13.9 ± 0.2	0.996 ± 0.002	0.016 ± 0.001
F3	0.567 ± 0.028	2.45 ± 0.05	0.992 ± 0.001	0.058 ± 0.004	0.952 ± 0.002	14.0 ± 0.3	0.997 ± 0.002	0.016 ± 0.001
F4	0.828 ± 0.013	2.34 ± 0.06	0.994 ± 0.000	0.055 ± 0.002	0.954 ± 0.003	13.5 ± 0.3	0.987 ± 0.006	0.016 ± 0.001
F8	0.768 ± 0.023	2.63 ± 0.04	0.989 ± 0.001	0.074 ± 0.003	0.946 ± 0.002	15.4 ± 0.3	0.996 ± 0.001	0.021 ± 0.001
F9	0.923 ± 0.012	2.39 ± 0.05	0.990 ± 0.000	0.051 ± 0.003	0.951 ± 0.001	13.5 ± 0.3	0.999 ± 0.000	0.014 ± 0.001
F10	0.956 ± 0.009	2.10 ± 0.03	0.992 ± 0.002	0.039 ± 0.001	0.947 ± 0.008	11.7 ± 0.1	0.996 ± 0.001	0.011 ± 0.000
F11	0.938 ± 0.004	2.30 ± 0.04	0.990 ± 0.003	0.047 ± 0.001	0.958 ± 0.002	12.9 ± 0.2	0.996 ± 0.001	0.013 ± 0.000

**Table 7:** Parameters of the Korsmeyer-Peppas kinetic model after fitting release data of different NLCs.

Formulation	Korsmeyer-Peppas		
	*R*^2^	*k*	*n*
F1	0.950 ± 0.009	15.3 ± 1.6	0.522 ± 0.027
F2	0.975 ± 0.009	9.76 ± 0.67	0.609 ± 0.017
F3	0.974 ± 0.007	9.39 ± 0.97	0.624 ± 0.027
F4	0.966 ± 0.001	10.1 ± 0.2	0.589 ± 0.012
F8	0.951 ± 0.004	13.0 ± 0.8	0.551 ± 0.015
F9	0.990 ± 0.004	7.53 ± 0.53	0.679 ± 0.015
F10	1.000 ± 0.000	5.65 ± 0.45	0.723 ± 0.024
F11	0.998 ± 0.001	7.5 0.13	0.686 ± 0.006

## 2 Experimental design, materials, and methods

### 2.1 Materials

OSH was purchased from Cadila Pharmaceuticals, India. Tristearin was supplied by Tokyo Chemical Industry, Japan. Phosal® 53MCT (Soybean lecithin 53% and medium-chain triglycerides) was received from Phospholipid GmbH, Germany. Polysorbate 80 (Tween® 80) was bought from Sigma-Aldrich, MO, USA. Glyceryl palmitostearate (Precirol® ATO 5), glyceryl monooleate (Peceol®), popylene glycol monolaurate (Lauroglycol® 90), propylene glycol monocaprylate (Capryol® 90), and caprylocaproyl polyoxyl-8 glycerides (Labrasol®) were provided by Gattefosse, France. Oleic acid and sesame oil were purchased from Daejung, South Korea. The HPLC grade water and acetonitrile were supplied by Avantor (PA, USA). Water was de-ionized and purified using the Milli-Q® purification system (Millipore, MA, USA). Other reagents were of analytical grade.

### 2.2 Preparation of NLCs using cold high-pressure homogenization method

NLCs were prepared using a modified cold high-pressure homogenization method (Fig. 1). Tristearin or Precirol® ATO 5 (solid lipid), Phosal® 53 MCT (liquid lipid), and the drug were dissolved in ethanol at 70±2 °C to prepare the lipid phase. Tween® 80 was dissolved in deionized water or various buffer solutions (pH 2.0–12.0) to prepare the aqueous phase. The aqueous phase was maintained at 4±2 °C. The lipid phase was transferred to the aqueous phase under magnetic stirring at 1,000 rpm. The mixture was continuously stirred at 4±2 °C for 2 h to obtain the coarse NLCs suspension. It was then subjected to high-pressure homogenization (Emulsiflex C3, Avestin, ON, Canada) at 0-4 °C. The homogenization condition (pressure and cycle number) was varied [5].

### 2.3 HPLC method for OSH analysis

An HPLC system (1260 Infinity, Agilent Technologies, CA, USA) consisted of a quaternary pump, an autosampler, and a UV detector. A reversed-phase column (Luna C18, 5 μm, 250 × 4.6 mm, Phenomenex, CA, USA) was used to separate samples. The mobile phase was acetonitrile/0.02 M acetate buffer pH 4.8 (40/60, V/V). The flow rate was 1.0 mL/min. The injection volume was 10 μl, and the detection wavelength was 310 nm.

### 2.4 Validation of HPLC method for OSH analysis

#### 2.4.1 Specificity

Specificity of the HPLC method was demonstrated by comparing the chromatograms of a blank sample and a standard sample. Fig. 2 shows chromatograms of the blank sample and OSH standard solution (10 μg/mL in the mobile phase). The typical chromatogram of OSH standard solution exhibits a sharp and well-resolved peak. There is no observation of visual peak in the blank sample chromatogram. The retention time of OSH peak is approximately 4.7 min. The peak symmetry is 0.80 and peak tail factor is 1.20. The chromatogram of a sample from release study (Fig. 2C) shows OSH peak at about 4.7 min with peak symmetry of 0.81 and peak tail factor of 1.20.

#### 2.4.2 System suitability

The system suitability of the developed HPLC method was demonstrated by various parameters including injection repeatability, peak tailing factor, and peak theoretical plate number. Chromatogram of OSH standard solution reveals a peak with the peak tailing factor of 1.20 and peak theoretical plates (USP) of more than 8890. Six injections of a standard solution (10 μg/mL) exhibits RSD = 0.4%. These results suggest that the HPLC system was suitable for OSH analysis.

#### 2.4.3 Linearity

A series of three replicates of five standard solutions with concentrations of 0.5, 1, 2, 5, and 10 μg/mL were analyzed using HPLC method. The relationship between OSH concentration and peak area is shown in Table 1A and Fig. 3. Peak areas of the calibration standard solutions were plotted in the Y-axis versus the standard concentrations. The HPLC method for analyzing OSH is linear in the concentration range of 0.5–10 μg/mL. The linear equation is y = 21.0250x-0.3804 and correlation coefficient (R^2^) is 0.9999, which demonstrates the strength of relationship between concentration of OSH and its corresponding peak area within the range 0.5–10 μg/mL in the developed HPLC method.

#### 2.4.4 Accuracy and precision

The percentage of variation coefficient (%CV) and percentage of recovery were calculated to demonstrate accuracy and precision, respectively. Three replicates at five different concentrations (0.5, 1, 2, 5, and 10 μg/mL) were used. The intraday accuracy and precision were analyzed within one day whereas the inter-day values were assessed over 3 different days. The results for accuracy and precision are illustrated in Table 1B. The precision of the HPLC assay ranges from 0.57 to 1.07% (intra-day) and from 0.43 to 2.84% (inter-day). The intra-day accuracy of the assay ranges from 98.06 to 101.88% and the inter-day accuracy ranges from 99.26 to 102.62%. The results reveals that the developed HPLC method is accurate and reliable for OSH determination.

### 2.5 OSH solubility in liquid lipids

OSH solubility in liquid lipids was determined as previously reported [6], [7]. An excess amount of OSH was dispersed in a glass vial containing a liquid lipid. The vials were vortex-mixed for dispersion of the drug. They were then sealed and shaken (50 rpm, 48 h) at 37 ± 0.5 °C using a shaking water bath. The samples were centrifuged at 14,000 rpm for 15 min, and the supernatants were collected and suitably diluted with ethanol. The samples were filtered using 0.45 μm membranes and analyzed by HPLC. Results of solubility studies were shown in Table 1.

### 2.6 Particle size analysis

PS and PI were determined by dynamic light scattering using a particle size analyzer (Zetasizer Nano-S90, Malvern Instruments, UK). After diluting 20 times with deionized water, samples were measured at 25 °C with a fixed detector angle of 90°.

### 2.7 EE and DL determination

The EE and DL were calculated by these equations:

EE (%) = OSH entrapped amount/Total OSH amount x 100.

DL (%) = OSH entrapped amount/(OSH entrapped amount + Lipid added amount) x 100.

OSH entrapped amount was calculated by subtracting the free OSH amount from the total OSH amount in the suspension [8]. The free OSH amount was determined by ultra-filtration centrifugation (14,000 rpm, 1 h) using a centrifugal filter unit (MWCO 10,000, Amicon® Ultra, Millipore, MA, USA). The free OSH in the filtrate was diluted with the mobile phase and analyzed using the HPLC method. The total OSH amount was determined by dissolving NLCs suspension in ethanol at 60 °C, diluting with the mobile phase, and quantifying by HPLC.

### 2.8 Fourier-transform infrared spectroscopy (FTIR)

An FTIR spectrophotometer (Tensor 27, Bruker, MA, USA) was used to analyze OSH, tristearin, Phosal® 53MCT, and the lyophilized F10 NLCs. FTIR spectra were recorded in the range of 500–4000 cm^−1^ with a resolution of 4 cm^−1^ for 16 times.

### 2.9 Mathematical modeling of release data

Following the *in vitro* drug release studies by dialysis bag method [9], data were fitted to various kinetic models (Zero-order, first-order, Higuchi, and Hixson-Crowell) using DDSolver, a recently developed program [10]. The mechanism of drug release was studied using the Korsmeyer-Peppas equation: *F* = *k.t*^*n*^, (*F* is the fraction of drug release, *k* is release constant, and *n* is diffusional exponent). The value of *n* could indicate the mechanism of drug release (*n* ≤ 0.5: Fickian diffusion, 0.5 < *n* ≤ 0.9: non-Fickian transport, and *n* ≥ 0.9: case-II transport) [11].
